# Drivers of Mosquito Microbiome Composition: Effects of Species, Locality, Season, and *Plasmodium* Infection

**DOI:** 10.1007/s00248-026-02801-7

**Published:** 2026-07-06

**Authors:** Marta Garrigós, Jesús Veiga, Mario Garrido, María José García-López, Manuel Morales-Yuste, Clotilde Marín, Jesús Recuero, María José Rosales, Isabel Moreno-Indias, Josué Martínez-de la Puente

**Affiliations:** 1 Estación Biológica de Doñana (EBD-CSIC), C/ Américo Vespucio, 26, 41092 Sevilla, Spain; 2 CIBER de Epidemiología y Salud Pública (CIBERESP), Madrid, Spain; 3 Departamento de Biología, Universidad Rey Juan Carlos (URJC), Tulipán s/n, 28933 Móstoles, Spain; 4 Instituto de Investigación en Cambio Global (IICG-URJC), Universidad Rey Juan Carlos (URJC), Tulipán s/n, 28933 Móstoles, Spain; 5 Instituto de Investigación Biomédica de Málaga y Plataforma en Nanomedicina (IBIMA Plataforma BIONAND), Málaga, Spain; 6 Department of Endocrinology and Nutrition, Virgen de la Victoria University Hospital, Málaga, Spain; 7 CIBER de Fisiopatología de la Obesidad y Nutrición (CIBEROBN), Madrid, Spain; 8 Faculty of Medicine, University of Málaga, Málaga, Spain; 9 Departamento de Parasitología, Universidad de Granada (UGR), Granada, Spain; 10 Veterinary and Conservation Department, Bioparc Fuengirola, Malaga, Spain

**Keywords:** *Aedes albopictus*, Avian malaria, *Culex pipiens*, Environmental variation, Invasive species, Microbiota, *Wolbachia*

## Abstract

Mosquito microbiota influences mosquito physiology and pathogen development, finally affecting their vectorial capacity. Identifying the factors that affect the composition of mosquito microbial communities in nature is essential for designing effective strategies to control vector-borne pathogens. Here, we used a 16 S rRNA metabarcoding approach to analyse the microbiome of 196 mosquito pools (four females per pool) of three common species: *Culex pipiens*, *Aedes albopictus*, and *Culiseta longiareolata*. Mosquitoes were collected from spring to autumn 2022 in five sampling localities of southern Spain. Mosquito bacterial alpha diversity was higher in *Cs. longiareolata* compared to *Cx. pipiens* and *Ae. albopictus*. In addition, beta diversity and the relative abundance of different bacterial taxa differed among mosquito species. *Wolbachia* dominated the bacterial community of *Cx. pipiens* and *Ae. albopictus*, but were virtually absent in *Cs. longiareolata*. Furthermore, using *Cx. pipiens* -the most extensively sampled species here- we further investigated differences in the microbiome composition according to sampling localities, seasons, and avian *Plasmodium* infection status. Locality and season affected the bacterial alpha and beta diversity, with mosquitoes collected in autumn from the Fuengirola locality showing a higher observed richness. Differences in beta diversity among localities and seasons could be, at least in part, influenced by differences in beta dispersion. The relative abundance of different taxa in *Cx. pipiens* varied by locality, season, and avian *Plasmodium* infection status. In sum, both intrinsic and environmental factors influence mosquito microbiome, yet the potential consequences for pathogen transmission should be further addressed. This study provides a comprehensive framework to understand the ecological drivers of wild mosquito microbiome, a key step for predicting vector-pathogen interactions and improving strategies for vector-borne disease control.

**Supplementary Information:**

The online version contains supplementary material available at https://doi.org/10.1007/s00248-026-02801-7.

## Introduction

Mosquitoes are vectors of deadly pathogens that affect humans, livestock and wildlife, such as viruses and parasites [1]. Mosquito bacterial microbiome (hereafter mosquito microbiome), defined as the genetic material of the mosquito bacterial microbiota, has become a central focus of research due to its profound influence on mosquito biology, including the interactions of mosquito vectors with pathogens [2].

Mosquito microbiota is mostly acquired during the aquatic larval stages being essential for their development [3]. For example, the reduction of bacterial load in aquatic habitats can delay development, limit growth, and increase larval mortality [4–6]. In adult mosquitoes, the microbiota composition mostly derived from that of the aquatic stage, although is further modulated after feeding on plant secretions or vertebrate hosts [7, 8]. This results in differences among developmental stages of mosquitoes, with larval microbiota showing a notably more diverse composition than that of adults [2]. The bacterial communities of adult mosquitoes also modulate their biology [2]. For example, antibiotic treatments increased the survival rate of adult mosquitoes [9, 10] but reduced egg production [11]. Thus, understanding the intrinsic and ecological drivers of mosquito microbiome in the wild is essential to identify their role in the transmission of pathogens relevant to human and animal health.

Among other factors, species identity highly determines differences among adult mosquitoes [12] which could be due to genetic factors that influence the host selection of certain bacterial taxa [13] and factors including the selection of larval breeding sites [14] and the vertebrate hosts of mosquitoes [15]. Moreover, environmental factors largely shape the mosquito bacterial community, as reflected in the microbiome of mosquitoes collected in different locations varying in the land use [16] and levels of urbanization [14]. Seasonality also modulates mosquito microbiome, with mosquitoes showing differences in their bacterial alpha diversity among seasons, being higher in summer than in spring [12], and in autumn than in summer [16]. However, these patterns could vary according to the environmental conditions of the geographical regions studied. Some authors reported higher bacterial alpha diversity in mosquitoes during the wet season [14, 17] while others found the opposite pattern [18], or even, no seasonal differences in mosquito microbiome [19]. Spatial and temporal variations in mosquito microbiome may be a consequence of different microclimates in the larval breeding waters, among other factors. Differences in temperature [20] and organic matter [21] found in different locations and seasons may determine the bacterial communities present in these habitats, finally affecting mosquito microbiota acquisition.

In addition, mosquito microbiota may directly affect the interaction of mosquitoes with pathogens [22], although the direction of the effect may depend on the bacterial taxa and/or the pathogen considered [23, 24]. Studies on the interaction between mosquitoes and *Plasmodium* parasites, the causative agent of malaria, reported a higher bacterial alpha diversity in infected *Anopheles* mosquitoes than uninfected ones [25], along with shifts in the relative abundance of particular bacterial taxa [25, 26]. However, Oliveira et al. [27] did not find significant differences in the microbiome alpha or beta diversity of *Plasmodium*-infected and uninfected *Nyssorhynchus darlingi* mosquitoes from Brazil. These studies are limited to human malaria parasites, and mostly in *Anopheles* mosquitoes, leaving other wildlife mosquito-*Plasmodium* systems, largely unexplored. In addition to humans, *Plasmodium* parasites also infect multiple vertebrates, including mammals, reptiles, and birds. Among them, avian malaria parasites are known to contribute to severe population declines and, even, local extinctions in bird species, as is the case of avian *Plasmodium relictum* in Hawaii [28]. However, in spite of the relevance of mosquitoes for its transmission and the role of mosquito microbiota modulating mosquito-pathogen interactions, studies on these wildlife systems are scarce. Here, we used 16 S rRNA gene metabarcoding to investigate patterns of variation in the bacterial microbiome of field collected mosquitoes in relation to species identity, locality and season of capture, and avian *Plasmodium* infection. First, we assessed differences in the bacterial communities among three common vector species relevant in the transmission of human and wildlife pathogens, including: *Culex pipiens*, *Aedes albopictus*, and *Culiseta longiareolata*. Second, focusing on *Cx. pipiens*, we assessed how seasonal variation, sampling locality, and avian *Plasmodium* infection relate to changes in its microbiome. Based on previous literature, we predicted differences in bacterial community diversity and composition among mosquito species [12] and, in the case of *Cx. pipiens*, across localities [15, 16] and seasons [12, 16]. In particular, locality was predicted to exert a stronger influence on the mosquito microbiome than season [14]. Finally, avian *Plasmodium* infection in mosquitoes may be associated with specific bacterial taxa, although the direction of these associations may vary according to the bacterium considered [25].

## Methods

### Mosquito Collection and Avian *Plasmodium* Detection

**Fig. 1:**
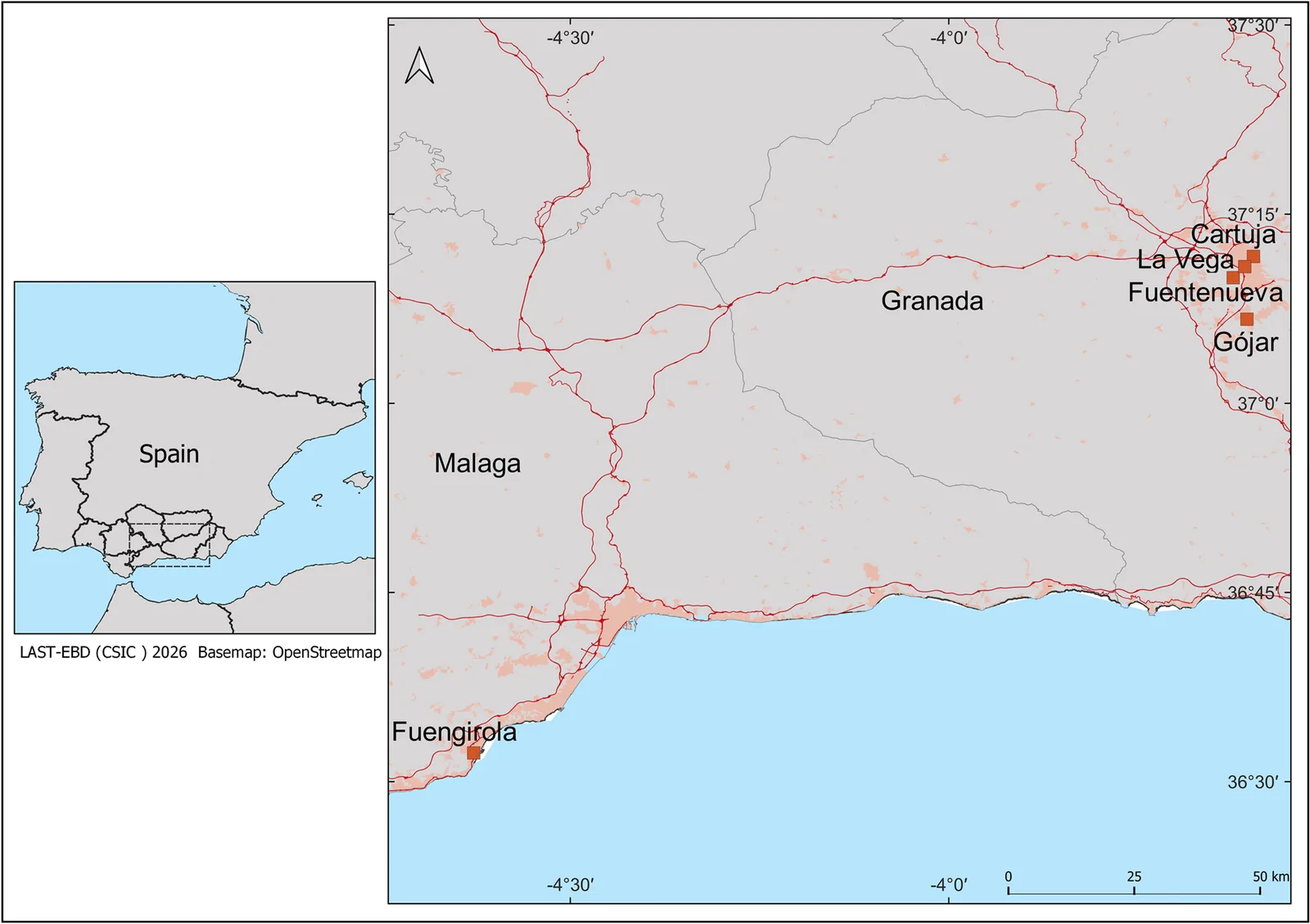
Study area with the five sampled localities in the provinces of Granada (upper right red squares) and Malaga (lower left red square) in southern Spain

Mosquitoes were trapped from May to November 2022 in five localities of the provinces of Granada (*n* = 4; La Vega; Fuentenueva campus; Cartuja campus, and Gójar) and Malaga (*n* = 1; the Bioparc zoological garden of Fuengirola), in southern Spain [29] (Fig. 1). Further details on mosquito collection are shown in Supplementary Material 1. Mosquito females of the same species, sampling session and locality, without any visible blood meal remnants in the abdomen, were grouped in pools of four individuals. The molecular screening for avian *Plasmodium* infection in mosquito pools was done in the context of a previous study [30]. In brief, Genomic DNA from each mosquito pool was extracted using the DNeasy ^®^ Blood & Tissue kit (Qiagen, Hilden, Germany) and the presence of avian *Plasmodium* DNA determined according to Hellgren et al. [31]. This technique consists in amplifying the cytochrome *b* gene of the parasite mitochondrial genome, with an expected amplicon length of approximately 480 bp (excluding primers). At least one negative control per 20 samples was included during DNA extractions and one negative control per plate for PCR reactions. Parasite detection was carried out at the mosquito pool level, therefore mosquito pools infected with *Plasmodium* may contain between one and four infected individuals. Positive samples were sequenced in both directions (STAB-VIDA, Lisbon, Portugal) and sequences edited using Geneious v2023.2.1. Parasite identity was confirmed by BLAST comparison (Basic Local Alignment Search Tool) with sequences deposited in GenBank and MalAvi [32] databases.

### Microbiome and Statistical Analyses

The microbiome of 196 mosquito pools were analysed, corresponding to 159 pools of *Cx. pipiens*, 23 of *Ae. albopictus* and 14 of *Cs. longiareolata*. The DNA from each mosquito pool was used to build the microbiome sequence libraries using the Ion 16 S Metagenomics^®^ kit (Thermofisher, Waltham, Massachusetts, USA), which contains primer pools to amplify multiple variable regions of the 16 S ribosomal RNA gene (V2, 3, 4, 6–7, 8 and 9). After generating amplicons, the Ion PlusTM Fragment Library Kit^®^ (Thermofisher, Waltham, Massachusetts, USA) was used to ligate barcoded adapters and synthesize the libraries. Barcoded libraries from all samples were pooled and templated on the automated Ion Chef system (Thermofisher, Waltham, Massachusetts, USA), followed by a 400 bp sequencing on the Ion S5 (Thermofisher, Waltham, Massachusetts, USA). For each sample, all reads including all the variable regions were provided as a single FASTA file and processed jointly during ASV inference. The 16 S rRNA gene PCR blanks did not amplify. Sequence quality was initially assessed using FastQC (ver. 0.12.1) [33] to inspect per-base quality profiles. Based on this inspection, reads were trimmed by removing the first 15 bp from the 5′ end (--p-trim-left 15) to eliminate low-quality and primer-derived bases, while no truncation was applied at the 3′ end (--p-trunc-len 0) to retain full-length reads. Denoising was performed using the DADA2 [34] denoise-pyro plugin in QIIME2 (ver. 2023.5.1) [35], which infers exact amplicon sequence variants (ASVs) while correcting sequencing errors. Chimera removal was carried out using DADA2’s built-in consensus method, applied by default during the denoising step. QIIME2 was also used to assign each Amplicon Sequence Variant (ASV) to a taxonomic group with an identity of 99% based on the SILVA database (ver. 138.1) [36]. Reads were filtered, removing singletons, reads that lacked taxonomic assignment below phylum level, and non-bacterial, chloroplast, and mitochondrial ASVs (Supplementary Table 1).

Further analyses were carried out in R (ver. 4.4.1) [37]. Packages *qiime2R* (ver. 0.99.6) [38] and *phyloseq* (ver. 1.48.0) [39] were used to import and filter the data, discarding samples with less than 1,000 reads (Supplementary Table 1). Then, we conducted statistical analyses to identify differences in bacterial alpha and beta diversity, as well as in taxa relative abundance among (1) species (*Cx. pipiens*, *Ae. albopictus* and *Cs. longiareolata*) and, in the case of *Cx. pipiens*, among (2) sampling localities (five sampling localities) and seasons, and according to the *Plasmodium* infection status (infected and uninfected mosquito pools). This analysis was restricted to *Cx. pipiens* because it was the only species with enough sample size and due to the fact that avian *Plasmodium* was only recorded in this species [30]. To account for seasonality, sampling sessions were grouped according to the season of the year: spring (sessions one and two, May 10-June 10), summer (sessions three to seven, June 23-September 27), and autumn (sessions eight to ten, October 5-November 22).

We estimate bacterial alpha diversity (within-sample diversity) of ASVs, including the observed richness and the Shannon index on samples rarefied to the minimum number of reads present in the samples (1,168 after data filtering), due to variability in the number of reads among samples. Normality of observed richness and Shannon index values was assessed visually and using the Shapiro-Wilk test. As normality assumptions were not met, differences in alpha diversity among mosquito species were evaluated using the Kruskal-Wallis test, followed by Dunn’s post hoc pairwise comparisons with Holm correction. For *Cx. pipiens*, differences in bacterial alpha diversity by sampling locality, season, and *Plasmodium* infection status were tested using additive Generalized Linear Models (GLMs) with a negative binomial (for the observed richness) or a gamma distribution (for the Shannon index). The distributions used in the GLMs were selected based on residual diagnostics, including the assessment of residual patterns, dispersion, and goodness-of-fit, to ensure an adequate representation of the underlying data structure. The sample size for some combinations of levels of the three factors was insufficient to fit models that included interactions; therefore, sampling locality, season, and *Plasmodium* infection status were included as explanatory variables in additive models. The individual contribution of each explanatory variable was estimated through the marginal R^2^ with the *glmm.hp* package (ver. 1.0–0) [40] and delta approximation. Pairwise comparisons were carried out using the Tukey test with the *emmeans* package (ver. 1.0–0) [41].

Beta diversity (between-sample diversity) was explored by a Principal Coordinate Analysis (PCoA) of the Bray-Curtis dissimilarity matrix on the ASVs relative abundances. The *vegan* package (ver. 2.6–10) [42] was used to perform a PERmutational Multivariate ANalysis Of VAriance (PERMANOVA) to test for differences between the centroids. The statistical significance of F-values obtained from the PERMANOVA was determined by comparison to 999 permutations. Pairwise comparisons were carried out using the *pairwiseAdonis* package (ver. 0.4.1) [43]. The beta dispersion among species, localities, seasons and infection statuses was checked using the ‘betadisper’ and ‘anova’ functions.

Differences in the relative abundance of bacterial families and genera among mosquito species and, for *Cx. pipiens*, among sampling localities and seasons and according to the *Plasmodium* infection status, were assessed using the ‘ancombc2’ function from *ancombc* package (ver. 2.6.0) [44], which fits a log-linear model with bias correction to estimate relative abundances. We fitted three models to identify bacteria showing differential abundances across sampling localities and seasons, and infection statuses for analyses on *Cx. pipiens*. In each model, the variable of interest was included as the main variable, and the other two were included as covariates to adjust for potential confounding effects. All variables were included in the models as fixed factors. Taxa with a prevalence below 10% were filtered out. Holm’s method was used to adjust the p-values for multiple testing (Supplementary Material 1). Plots were made using the *ggplot2* (v. 3.5.1) [45] and *ggvenn* [46] packages.

## Results

After quality filtering, the final dataset available for the microbiome analyses corresponded to 161 mosquito pools, including 131 *Cx. pipiens* pools, 18 *Ae. albopictus* pools and 12 *Cs. longiareolata* pools (Supplementary Table 1; Table S1). The dataset included a total of 14,820,507 reads, ranging from 1,168 to 444,952 per pool (average = 92,052.83).

### Microbiome Differences Among Mosquito Species

**Fig. 2:**
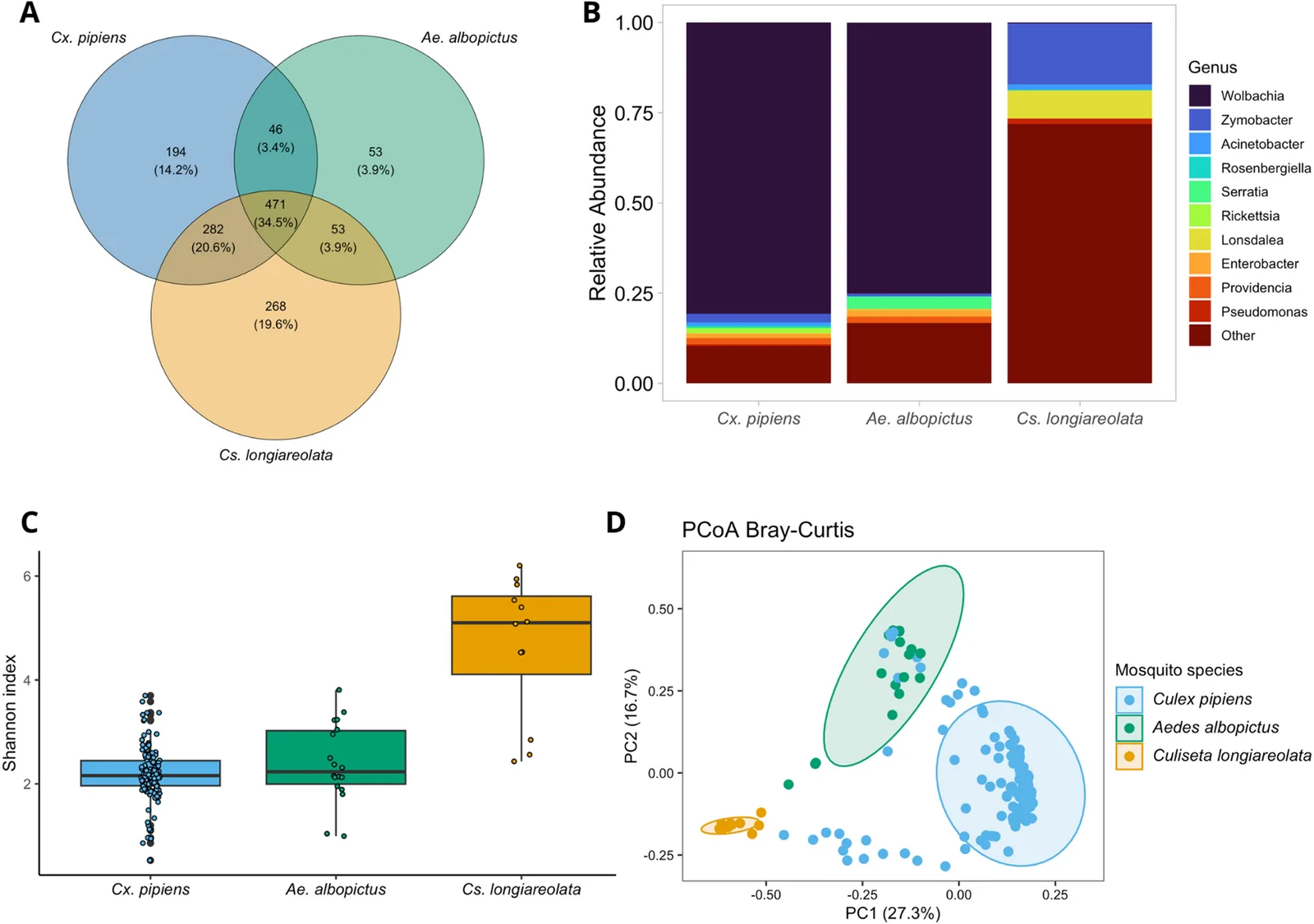
Mosquito bacterial microbiome of three common species in southern Spain: *Culex pipiens*, *Aedes albopictus* and *Culiseta longiareolata*. (**A**) Overlapping and unique bacterial genera among mosquito species. (**B**) Relative abundance of the 10 most abundant bacterial genera in *Cx. pipiens*, *Ae. albopictus* and *Cs. longiareolata*. The remaining bacterial genera are grouped under the “Other” category. (**C**) Differences in Shannon diversity index in the microbiome of *Cx. pipiens*, *Ae. albopictus* and *Cs. longiareolata*. The horizontal lines represent the median Shannon index while the boxes delimit the upper (75%) and lower (25%) quartiles, the vertical lines go from the lower and upper quartiles to the minimum or maximum values, dots represent the value per each mosquito pool. (**D**) Principal Co-ordinates Analysis (PCoA) for Bray-Curtis distance matrix of ASVs (relative abundance) of the bacterial communities of *Cx. pipiens*, *Ae. albopictus* and *Cs. longiareolata*. PC1 and PC2 axes refer to the percentage of variation explained by each of the two selected main coordinate axes. Ellipses represent 95% confidence intervals around group centroids

Overall, 16,373 ASVs were identified, classified into 34 different phyla and, at least, 95 classes, 255 orders, 480 families, and 1,367 genera. Of these, 471 (34.5%) genera were present in all three mosquito species, while 194 (14.2%), 53 (3.9%), and 268 (19.6%) genera were exclusively found in *Cx. pipiens*, *Ae. albopictus*, and *Cs. longiareolata*, respectively (Fig. 2A). The most abundant phyla were Proteobacteria (average = 96.49%, SD = 0.08) and Bacillota (formerly Firmicutes) (average = 1.31%, SD = 0.03). At genus level, the bacterial microbiome of *Cx. pipiens* and *Ae. albopictus* were dominated by *Wolbachia* (family *Anaplasmataceae*) with a mean relative abundance of 83.12% (SD = 0.24) and 76.20% (SD = 0.27), respectively. No bacteria clearly dominated *Cs. longiareolata* microbiome. The most abundant genera in *Cs. longiareolata* were *Zymobacter* (average = 7.80%, SD = 0.23) and *Lonsdalea* (average = 3.17%, SD = 0.09) (Fig. 2B).

Bacterial richness (chi-squared = 42.067, df = 2, *P* < 0.001) and Shannon index (chi-squared = 28.126, df = 2, *P* < 0.001) significantly varied among species. The observed richness was higher in *Cs. longiareolata* (Z = -5.563, P adjusted < 0.001) and *Ae. albopictus* (Z = -3.878, P adjusted < 0.001) compared to *Cx. pipiens* (Fig S1), and the Shannon index was higher in *Cs. longiareolata* compared to both *Cx. pipiens* (Z = -5.279, P adjusted < 0.001) and *Ae. albopictus* (Z = 3.572, P adjusted < 0.001) (Fig. 2C). The PERMANOVA revealed significant differences in Bray-Curtis structure by species (PERMANOVA: F = 31.660, df = 2, *P* = 0.001). In the PCoA ordination (Fig. 2D), species centroids were clearly separated, with no overlap among group ellipses, suggesting distinct bacterial communities across species. However, there were significant differences in beta dispersion among mosquito species (F = 12.189, *P* < 0.001), thus differences in beta diversity may be affected, at least in part, by differences in within-species dispersion. The pairwise comparison showed differences between the three mosquito species (F_ae−cx_ = 29.035; F_ae−cs_ = 14.888; F_cx−cs_ = 39.706, d = 2, all *P* = 0.001). Most significant differences in bacterial relative abundances occurred between *Cs. longiareolata* and *Ae. albopictus* (17 families and 20 genera), followed by *Cx. pipiens* and *Cs. longiareolata* (16 families and 13 genera), and *Cx. pipiens* and *Ae. albopictus* (8 families and 8 genera) (Supplementary Table 2).

### Effects of Sampling Locality and Season, and Avian *Plasmodium* Infection

**Fig. 3:**
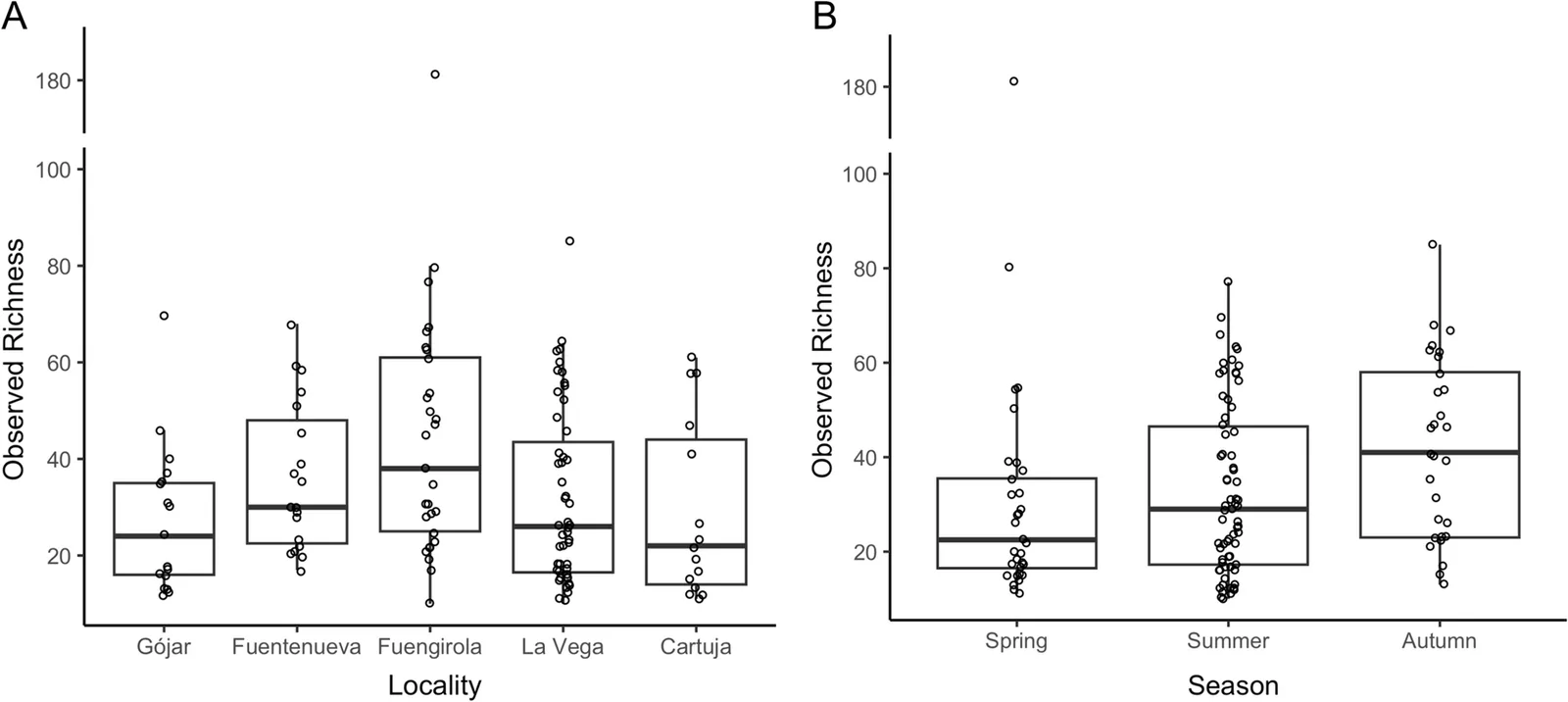
Observed richness of the bacterial microbiome of *Culex pipiens* female mosquito pools among localities (**A**) and seasons (**B**). The horizontal lines represent the median observed richness while boxes delimit the upper (75%) and lower (25%) quartiles, and the vertical lines go from the lower and upper quartiles to the minimum or maximum values, dots represent the value per each mosquito pool

Of the 131 *Cx. pipiens* pools included in the analyses, 27 samples harboured avian *Plasmodium* parasites while 104 were diagnosed as uninfected. The bacterial alpha diversity, including both the observed richness and the Shannon index, significantly varied by sampling locality and season, but not by infection status. The three additive effects explained the 17.82% and the 8.90% of the observed richness and the Shannon index, respectively. Of the total variation explained by the model, most part can be explained by sampling locality (observed richness = 62.96%, Shannon index = 80.25%), followed by season (observed richness = 30.42%, Shannon index = 16.84%). Pairwise comparisons showed a higher observed richness in mosquitoes from Fuengirola compared to those from Gójar, La Vega, and Cartuja, and in mosquitoes captured in autumn than in those captured in spring and summer (Fig. 3; Table 1). In addition, Fuentenueva showed a higher Shannon index than Gójar (Table 1). Similarly, the PERMANOVA showed significant differences in Bray-Curtis structure among sampling localities and seasons, but not by infection status. In particular, locality explained most part of the variation in the general bacterial composition (PERMANOVA: R2 = 0.075, *P* = 0.003), followed by season (PERMANOVA: R2 = 0.026, *P* = 0.001). Significant differences in beta dispersion between localities (ANOVA: F = 2.470, *P* = 0.048) and seasons (ANOVA: F = 4.827, *P* = 0.010) suggest that the observed differences in beta diversity are, at least in part, driven by differences in within-group dispersion, rather than reflecting true differences in bacterial community composition. Pairwise comparisons showed differences among Gójar, Fuentenueva and La Vega, between Fuentenueva and Fuengirola, and between La Vega and Cartuja. In the case of season, the beta diversity index differed between spring and autumn.

**Table 1:** Pairwise comparisons of bacterial alpha and beta diversity in *Culex pipiens* by avian *Plasmodium* infection, locality, and season. nbGLM = Generalized Linear Model with negative binomial distribution. GLM-G = Generalized Linear Model with gamma distribution

Diversity	Metric	Model (pairwise test)	Factor (df)	Comparison	Estimate	*P*
Alpha	Observed richness	nbGLM (Tukey)	Infection status (1)	Infected vs. uninfected	z = 1.367	0.453
			Locality (4)	Gójar vs. Fuentenueva	z = -1.506	0.558
				Gójar vs. Fuengirola	z = -3.123	**0.015**
				Gójar vs. La Vega	z = -0.681	0.961
				Gójar vs. Cartuja	z = 0.254	0.999
				Fuentenueva vs. Fuengirola	z = -1.608	0.492
				Fuentenueva vs. La Vega	z = 1.176	0.765
				Fuentenueva vs. Cartuja	z = 1.726	0.412
				Fuengirola vs. La Vega	z = 3.345	**0.007**
				Fuengirola vs. Cartuja	z = 3.375	**0.007**
				La Vega vs. Cartuja	z = 0.958	0.874
			Season (2)	Spring vs. Summer	z = -0.485	0.878
				Spring vs. Autumn	z = -2.718	**0.018**
				Summer vs. Autumn	z = -2.738	**0.017**
	Shannon index	GLM-G (Tukey)	Infection status (1)	Infected vs. uninfected	t = 0.296	0.766
			Locality (4)	Gójar vs. Fuentenueva	t = -2.975	**0.029**
				Gójar vs. Fuengirola	t = -1.911	0.317
				Gójar vs. La Vega	t = -1.526	0.548
				Gójar vs. Cartuja	t = -0.740	0.947
				Fuentenueva vs. Fuengirola	t = 1.375	0.645
				Fuentenueva vs. La Vega	t = 2.120	0.218
				Fuentenueva vs. Cartuja	t = 2.123	0.217
				Fuengirola vs. La Vega	t = 0.671	0.962
				Fuengirola vs. Cartuja	t = 1.051	0.831
				La Vega vs. Cartuja	t = 0.583	0.977
			Season (2)	Spring vs. Summer	t = -0.174	0.983
				Spring vs. Autumn	t = -1.292	0.403
				Summer vs. Autumn	t = -1.355	0.368
Beta	Bray- Curtis	PERMANOVA	Infection status (1)	Infected vs. uninfected	F = 0.575	0.891
			Locality (4)	Gójar vs. Fuentenueva	F = 3.501	**0.003**
				Gójar vs. Fuengirola	F = 2.099	0.052
				Gójar vs. La Vega	F = 3.716	**0.004**
				Gójar vs. Cartuja	F = 1.244	0.275
				Fuentenueva vs. Fuengirola	F = 2.353	**0.008**
				Fuentenueva vs. La Vega	F = 4.713	**0.001**
				Fuentenueva vs. Cartuja	F = 1.832	0.054
				Fuengirola vs. La Vega	F = 1.742	0.089
				Fuengirola vs. Cartuja	F = 1.232	0.254
				La Vega vs. Cartuja	F = 2.434	**0.027**
			Season (2)	Spring vs. Summer	F = 1.252	0.219
				Spring vs. Autumn	F = 2.727	**0.006**
				Summer vs. Autumn	F = 1.724	0.084

**Fig. 4:**
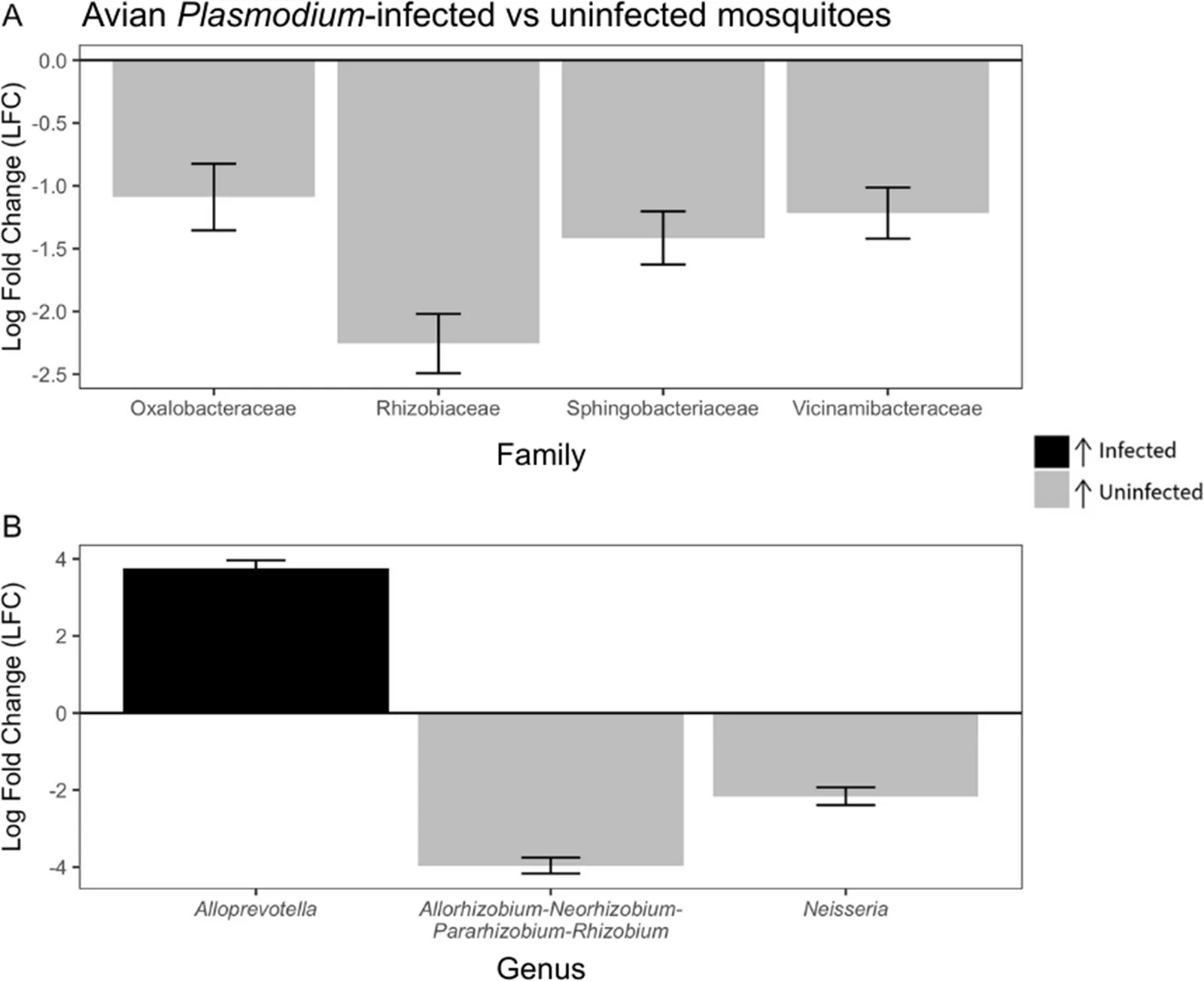
Differences in the relative abundance of bacterial families (**A**) and genera (**B**) in *Culex pipiens* pools infected and uninfected with avian *Plasmodium*. The y-axis represents the Log Fold Change (LFC), quantifying the magnitude of differences in relative abundance of each taxon between infected and uninfected pools. Bacterial genera showing a higher relative abundance in infected *Cx. pipiens* pools are shown in black, while those with a higher relative abundance in uninfected *Cx. pipiens* pools are represented in grey. Error bars represent standard errors

Differences in the relative abundance of bacterial taxa according to sampling locality, season and *Plasmodium* infection status are detailed in Supplementary Table 3. The sampling locality of Gójar showed differences in taxa relative abundances compared to Fuentenueva, Fuengirola, and La Vega. In addition, there were differences in taxa relative abundance between La Vega and Fuentenueva, and between Cartuja y Fuengirola. Regarding season, most differences in the relative abundance of bacterial taxa in mosquito pools were found between summer and the other two seasons, whereas spring and autumn showed fewer differences between them. Finally, the microbiome of *Plasmodium* infected *Cx. pipiens* pools had a higher relative abundance of bacteria of the genus *Alloprevotella*, and a lower relative abundance of the families *Rhizobiaceae*, *Sphingobacteriaceae*, *Vicinamibacteraceae* and *Oxalobacteraceae*, and the genera *Allorhizobium-Neorhizobium-Pararhizobium-Rhizobium* and *Neisseria*, than pools of uninfected mosquitoes (Fig. 4).

## Discussion

Mosquito bacterial communities play a key role in modulating mosquito biology, including development, survival and reproduction, as well as pathogen development and transmission, supporting the necessity of identifying those intrinsic and environmental factors shaping mosquito microbiome in the wild. Previous studies have primarily focused on specific mosquito species and regions, with a main interest on major vectors of pathogens with public health relevance [25, 26]. However, there is still a large knowledge gap about the bacterial community composition of common mosquito species in Europe, including currently spreading invasive species such as *Ae. albopictus*. Our results add valuable information in this respect by the characterisation and comparison of the bacterial microbiome of three wild common mosquito species cohabiting in the same area. In addition, we provide support for the geographical and temporal variation of mosquito microbiome at the intraspecific level as well as according to the interactions with wildlife mosquito-borne pathogens.

### Differences in Mosquito Microbiome Between Species

Species identity is a key component explaining the composition of mosquito microbiome [12, 47], a pattern supported by our results. *Culiseta longiareolata* had a higher bacterial evenness -estimated by the Shannon index- than *Cx. pipiens* and *Ae. albopictus*. This may be explained by the greater bacterial richness observed in *Cs. longiareolata* with non-clearly dominating bacteria genus, which contrasts with *Cx. pipiens* and *Ae. albopictus* in which *Wolbachia* dominated. The dominance of *Wolbachia* has been reported in different species of *Culex* and *Aedes* worldwide, including *Cx. pipiens* and *Ae. albopictus* [48], as well as its total absence in *Cs. longiareolata* [49]. These differences in *Wolbachia* among mosquito species inhabiting the same geographical area may reflect differences in: host genetic compatibility, exposure for the acquisition of this bacterium, microbiome composition that limit the establishment or vertical transmission of the symbiont [50]. In addition to *Wolbachia*, the three mosquito species showed a notable proportion of exclusive bacterial taxa and differences in the relative abundance of some of them. Similarly, different genetic background among mosquito species may imply differences in immune responses and gut environment, which may explain differences in their bacterial communities [51]. Alternatively, interspecific differences in larval breeding-site preferences and adult behaviour, including variation in predominant blood-feeding sources [52], may also contribute to the observed patterns. Mosquito microbiota is strongly shaped by the microbial communities present in the larval environment [2] and may additionally be influenced by the vertebrate bloodmeal sources of mosquitoes [15]. These differences in the bacterial communities among species may influence mosquito development success [2], reproduction output [53], survival rates [54], resistance to insecticides [55], and pathogen development [22], finally affecting their vectorial capacity [54]. We also detected differences in bacterial community variability among species. To rule out the possible effect of differences in sample size between the mosquito species on the beta dispersion of their bacterial communities, we repeated these analyses using a random subset that contained the same number of mosquito groups per species, obtaining similar results. We hypothesised that the within-species variability may reflect differences in geographic distribution, feeding sources, and diversity of breeding habitats, which could promote increased heterogeneity in microbial community composition of mosquitoes [56, 57]. 

### Effects of Sampling Locality and Season on *Culex pipiens* Microbiome

The bacterial microbiome of *Cx. pipiens* was associated with two extrinsic factors, sampling locality and season, which affected the bacterial alpha and beta diversity and general bacteria composition [12, 17, 58]. Specifically, locality explained a higher percentage of the variability in bacterial diversity, than season. Sampling locality is a key component explaining differences in the microbiome composition of mosquitoes, with insects sampled in the same locality showing more similar microbiomes [47]. This pattern could be explained based on differences in the bacterial communities found in the breeding sites from different areas [59], as mosquitoes acquire most part of their microbiota during the larval stage from their breeding waters [2]. In addition, habitat characteristics, including land use [16] and the level of urbanization [14], may determine the composition of mosquito microbiome. All of these factors may explain, at least in part, the variation in *Cx. pipiens* mosquitoes accounted in our study.

In addition to geographical differences, we identified changes in the composition of mosquito bacterial community along the study period. We observed a higher bacterial alpha diversity in mosquitoes collected during autumn compared to those sampled in spring and summer, in accordance with a previous study developed in United States [16]. In southern Spain, spring is characterised by a decrease in humidity while the temperatures rise, while summer is considered a hot and dry season and during autumn temperatures typically decrease with an increasing humidity. These differences in temperature and humidity may determine differences in the microbiome composition of mosquitoes. For example, the bacterial alpha diversity of mosquitoes decreases under low- or high temperature stress [20, 60], and changes in beta diversity and the relative abundance of certain bacteria were associated to temperature variation [20]. These effects could be also due to differences in the relative abundance of specific bacteria along the study period. For instance, *Methylobacterium* and *Acinetobacter* showed a higher relative abundance in mosquitoes collected in autumn than those sampled in summer, two bacteria which were previously associated with the wet season in other regions [10, 17]. However, contrasting patterns were found in mosquitoes captured in other regions, showing either a higher [17] or a lower [14, 18] bacterial alpha diversity in mosquitoes collected during the wet season with respect to those sampled during the dry season. Climatic differences between study areas may account for the contrasting patterns previously found, highlighting the complexity of the interactions among environmental and intrinsic factors that shape mosquito microbiome. Nevertheless, season and locality should not be interpreted as fully independent ecological axes in our sampling design. Differences in climatic and environmental conditions among localities, including sampling localities of Malaga and Granada provinces, might covary with seasonal patterns and thus contribute to part of the observed variation in bacterial communities.

Given the key role of mosquito-associated microbiota in modulating vectorial capacity, spatial-temporal variation in the microbiome of *Cx. pipiens* may have important ecological consequences for the transmission of the pathogens vectored by this species, such as avian *Plasmodium*. Avian malaria parasites infect a wide range of bird species and have detrimental effects on host health [61], explaining the population decline of common species such as the house sparrow (*Passer domesticus*) [62]. Consequently, heterogeneous infection patterns among bird populations could be driven by differences between localities and seasons of intrinsic factors of mosquito populations [63], including their bacterial community. For example, seasonal variation in the prevalence of the zoonotic West Nile virus has been linked to changes in *Wolbachia* loads in mosquitoes [13]. However, whether avian *Plasmodium* exhibits similar microbiome-mediated seasonal or spatial patterns remains unknown.

### *Culex pipiens* Microbiome Associated to Avian *Plasmodium* Infection

We did not find significant differences in mosquito bacterial alpha or beta diversity according to *Plasmodium* infection, in line with previous studies [26, 27]. However, Bassene et al. [25] observed a higher bacterial alpha diversity in the human assemblage *Plasmodium falciparum* - *Anopheles* spp. In spite of the lack of differences in overall bacterial community, we found changes in the relative abundance of specific bacterial taxa associated with *Plasmodium* infection status. A similar pattern was previously recorded [25, 26, 58]. For instance, using field trapped mosquitoes, we found a lower relative abundance of bacteria of the genera *Rhizobium* and *Neisseria* in infected pools. Similarly, mosquitoes infected with *Ascogregorina taiwanensis* also had a relatively lower abundance of *Rhizobium* [58]. Differences in certain bacteria strains according to the infection status could be due to their protective role against *Plasmodium* spp. in mosquitoes [64, 65], which could be mediated by an up-regulation of immune genes and anti-*Plasmodium* factors [65]. In contrast, the genus *Alloprevotella* exhibited a higher relative abundance in *Plasmodium*-infected pools, suggesting an opposite pattern. Overall, our findings highlight several bacterial taxa that may potentially influence, either positively or negatively, avian malaria infections. However, the correlative approach of our study prevents us to establish the causality of these associations, being necessary experimental studies using, for example, gnotobiotic mosquitoes, to elucidate such interactions.

Our study has some limitations. For example, we analysed the bacterial community of mosquitoes grouped in pools of four females, and therefore, positive samples may contain a variable number (from one to four) of infected individuals. This could dilute the potential differences between avian *Plasmodium*-infected and uninfected mosquitoes. Therefore, we would expect differences associated to avian malaria infection to be greater than what we observed here. In addition, *Plasmodium*-positive mosquitoes had taken an avian blood meal, whereas we are unaware of whether uninfected mosquitoes had fed on blood at some point. To minimize this potential source of bias, mosquitoes included in this study showed no blood remnants in the abdomen, indicating that any previous blood meal had occurred, at least, 48 h before collection [66]. This may allow mosquito microbiota to return to its baseline composition [67]. Furthermore, as this study uses mosquitoes captured in the field, we did not control for other potential confounding variables such as mosquito age, microhabitat characteristics, or sugar feeding sources. Finally, because 16 S rRNA amplicon data are compositional, changes in the relative abundance of individual taxa should be interpreted as shifts in community composition rather than as absolute increases or decreases in bacterial abundance.

## Conclusion

Species identity largely shapes the diversity and composition of the bacterial community of mosquitoes from southern Spain. While the bacterial microbiomes of *Cx. pipiens* and *Ae. albopictus* are dominated by *Wolbachia*, *Cs. longiareolata* lacks this endosymbiont. The microbiome diversity of *Cx. pipiens* is primarily structured by locality, followed by season, with no detectable effect of avian *Plasmodium* infection. Nevertheless, specific bacterial taxa are associated with avian *Plasmodium* infection status at the mosquito pool-level. Given the central role of the mosquito microbiota in modulating the insect vectorial capacity, interspecific and spatial and temporal variation in bacterial communities may have important epidemiological implications. Future experimental studies are needed to determine the causality of these associations and identify the underlying mechanisms mediating these interactions.

## Supplementary Information

Below is the link to the electronic supplementary material.

Supplementary File 1 (XLSX 22.5 KB)

Supplementary File 2 (XLSX 18.5 KB)

Supplementary File 3 (XLSX 17.5 KB)

Supplementary File 4 (DOCX 142 KB)

## Data Availability

Data analysed in the present study are publicly available at: https://doi.org/10.20350/digitalCSIC/18469.
