# Supplementary material for: Drivers of Mosquito Microbiome Composition: Effects of Species, Locality, Season, and Plasmodium Infection
Source: Microb Ecol. 2026 Jul 6;89:179. doi: 10.1007/s00248-026-02801-7 (PMC13616892; doi:10.1007/s00248-026-02801-7)
Supplement: Supplementary file 1 [file 248_2026_2801_MOESM4_ESM.docx]

**Drivers of mosquito microbiome composition: effects of species, locality, season, and *Plasmodium* infection**

Marta Garrigós^1,2*^, Jesús Veiga^1,2^, Mario Garrido^3,4^, María José García-López^5,6,7,8^, Manuel Morales-Yuste^9^, Clotilde Marín^9^, Jesús Recuero^10^, María José Rosales^9^, Isabel Moreno-Indias^5,6,7^, Josué Martínez-de la Puente^1,2^

1.	Estación Biológica de Doñana (EBD-CSIC), Sevilla, Spain.

2.	CIBER de Epidemiología y Salud Pública (CIBERESP), Madrid, Spain.

3.	Departamento de Biología, Universidad Rey Juan Carlos (URJC), Tulipán s/n, 28933 Móstoles, Spain

4.	Instituto de Investigación en Cambio Global (IICG-URJC), Universidad Rey Juan Carlos (URJC), Tulipán s/n, 28933 Móstoles, Spain.

5.	Instituto de Investigación Biomédica de Málaga y Plataforma en

Nanomedicina (IBIMA Plataforma BIONAND), Málaga, Spain.

6.	Department of Endocrinology and Nutrition, Virgen de la Victoria University

Hospital, Málaga, Spain

7.	CIBER de Fisiopatología de la Obesidad y Nutrición (CIBEROBN), Madrid, Spain.

8	Faculty of Medicine, University of Málaga, Málaga, Spain

9.	Departamento de Parasitología, Universidad de Granada (UGR), Granada, Spain.

10.	Veterinary and Conservation Department, Bioparc Fuengirola, Malaga, Spain

* Corresponding author. Marta Garrigós. Estación Biológica de Doñana (EBD-CSIC), C/ Américo Vespucio, 26, 41092, Seville, Spain. E-mail: [marta.garrigos@ebd.csic.es](mailto:marta.garrigos@ebd.csic.es)

1. **Supplementary Tables and Figures**

**Table S1.** Total number of pooled samples included in the final analysis for each mosquito species, *Culex pipiens*, *Aedes albopictus* and *Culiseta longiareolata*, in each locality and season. The number of pools infected with avian *Plasmodium* is shown in brackets, where applicable.

| **Locality** | **Season** | ***Culex pipiens*** | ***Aedes albopictus*** | ***Culiseta longiareolata*** | **Total** |
| --- | --- | --- | --- | --- | --- |
| **Cartuja** | **Spring** | 0 | 0 | 1 | 1 |
|  | **Summer** | 10 (1) | 8 | 0 | 18 |
|  | **Autumn** | 5 (1) | 1 | 0 | 6 |
| **Fuengirola** | **Spring** | 5 (1) | 0 | 0 | 5 |
|  | **Summer** | 19 (2) | 2 | 0 | 21 |
|  | **Autumn** | 5 | 0 | 0 | 5 |
| **Fuentenueva** | **Spring** | 6 (2) | 0 | 0 | 6 |
|  | **Summer** | 9 (2) | 2 | 0 | 11 |
|  | **Autumn** | 4 | 0 | 0 | 4 |
| **Gójar** | **Spring** | 2 | 0 | 6 | 8 |
|  | **Summer** | 11 (6) | 5 | 0 | 16 |
|  | **Autumn** | 4 (2) | 0 | 0 | 4 |
| **La Vega** | **Spring** | 19 (4) | 0 | 4 | 23 |
|  | **Summer** | 21 (5) | 0 | 1 | 22 |
|  | **Autumn** | 11 (1) | 0 | 0 | 11 |
| **Total** |  | **131 (27)** | **18** | **12** | **161** |

**Fig S1.** Differences in observed richness in the microbiome of *Cx. pipiens*, *Ae. albopictus* and *Cs. longiareolata*. The horizontal lines represent the median observed richness while the boxes delimit the upper (75%) and lower (25%) quartiles, the vertical lines go from the lower and upper quartiles to the minimum or maximum values, dots represent the value per each mosquito pool.

2. **Extended material and methods**
   2.1. Mosquito collection

Mosquitoes were trapped from May to November 2022 in five localities of the provinces of Granada (n = 4; La Vega, 37°09′57.6"N; 3°37′27.6"W; Fuentenueva campus, 37°10′50.4"N; 3°36′32.7"W; Cartuja campus, 37°11′38.7"N; 3°35′51.2"W and Gójar, 37°06′40.2"N; 3°36′22.4"W) and Malaga (n = 1; the Bioparc zoological garden of Fuengirola, 36°32′16.7"N; 4°37′39.5"W), in southern Spain. Mosquitoes were collected with four traps per sampling locality and session, two Blacklight (UV)-CDC Miniature traps (Centers for Disease Control and Prevention, Atlanta, GA, USA) and two Biogents (BG)-Sentinel-2 traps (Biogents, Regensbourg, Germany), the last supplemented with dry ice as a source of CO_2_ and BG-Lure mosquito attractant (see Garrido et al. (2024) for details of the sampling procedure). One additional CDC-UV trap was used in the zoological garden to maximize the number of mosquitoes captured in this area. Each sampling session was carried out for approximately 24 hours, except at the biological garden, where traps operated from 7:00 p.m. to 10:00 a.m. (local time) minimizing the hours with higher affluence of visitors. Overall, 10 sampling sessions were conducted, separated every 2–3 weeks. Collected mosquitoes were frozen and transported to the laboratory on dry ice, where they were morphologically identified (Schaffner et al. 2001) and, subsequently, stored at –80 °C. Mosquito females of the same species, sampling session and locality, without any visible blood meal remnants in the abdomen, were grouped in pools of 4 individuals (irrespective of the trap in which they were collected).

2.2. *ANCOMBC2 models*

Differences in taxa relative abundance by species:

ancombc2(data = physeq_filt, tax_level ="**Family"/"Genus"*,** **fix_formula = "species"**, p_adj_method = "holm", pseudo_sens = TRUE, prv_cut = 0.10, s0_perc = 0.05, **group = "species"**, struc_zero = TRUE, neg_lb = FALSE, alpha = 0.05, n_cl = 2, global = FALSE, pairwise = TRUE, dunnet = FALSE, trend = FALSE, iter_control = list(tol = 1e-5, max_iter = 20, verbose = FALSE), em_control = list(tol = 1e-5, max_iter = 100), lme_control = NULL, mdfdr_control = list(fwer_ctrl_method = "holm", B = 100), trend_control = NULL)

Differences in taxa relative abundance by locality:

ancombc2(data = physeq_filt, tax_level ="**Family"/"Genus"*, fix_formula = "locality+season+infection"**, p_adj_method = "holm", pseudo_sens = TRUE, prv_cut = 0.10, s0_perc = 0.05, **group = "locality"**, struc_zero = TRUE, neg_lb = FALSE, alpha = 0.05, n_cl = 2, global = FALSE, pairwise = TRUE, dunnet = FALSE, trend = FALSE, iter_control = list(tol = 1e-5, max_iter = 20, verbose = FALSE), em_control = list(tol = 1e-5, max_iter = 100), lme_control = NULL, mdfdr_control = list(fwer_ctrl_method = "holm", B = 100), trend_control = NULL)

Differences in taxa relative abundance by season:

ancombc2(data = physeq_filt, tax_level ="**Family"/"Genus"*,** **fix_formula = "season+locality+infection",** p_adj_method = "holm", pseudo_sens = TRUE, prv_cut = 0.10, s0_perc = 0.05, **group = "season"**, struc_zero = TRUE, neg_lb = FALSE, alpha = 0.05, n_cl = 2, global = FALSE, pairwise = TRUE, dunnet = FALSE, trend = FALSE, iter_control = list(tol = 1e-5, max_iter = 20, verbose = FALSE), em_control = list(tol = 1e-5, max_iter = 100), lme_control = NULL, mdfdr_control = list(fwer_ctrl_method = "holm", B = 100), trend_control = NULL)

Differences in taxa relative abundance by avian *Plasmodium* infection:

ancombc2(data = physeq_pipiens, tax_level ="**Family"/"Genus"***, **fix_formula =** **"infection+locality+season"**, p_adj_method = "holm", pseudo_sens = TRUE, prv_cut = 0.10, s0_perc = 0.05, group = **"infection",** struc_zero = TRUE, neg_lb = FALSE, alpha = 0.05, n_cl = 2, dunnet = FALSE, trend = FALSE, iter_control = list(tol = 1e-5, max_iter = 20, verbose = FALSE), em_control = list(tol = 1e-5, max_iter = 100), lme_control = NULL, mdfdr_control = list(fwer_ctrl_method = "holm", B = 100), trend_control = NULL)

* A model was fitted at the family level using the "Family" argument and another at the genus level using the "Genus" argument

**References**

Garrido, M., Veiga, J., Garrigós, M., Morales-Yuste, M., Recuero-Gil, J., & Martínez-de la Puente, J. (2024). *Aedes albopictus* in a recently invaded area in Spain: effects of trap type, locality, and season on mosquito captures. Scientific Reports, 14(1), 2131. <https://doi.org/10.1038/s41598-024-52040-4>

Schaffner F., Angel Guy, Geoffroy Bernard, Hervy Jean-Paul, Rhaiem A., Brunhes Jacques. (2001). Les moustiques d’Europe: logiciel d'identification et d'enseignement. The mosquitoes of Europe: an identification and training programme. Paris (FRA); Montpellier: IRD; EID, 1 CD ROM. (Didactiques).
